# Engineering *Shewanella oneidensis* enables xylose-fed microbial fuel cell

**DOI:** 10.1186/s13068-017-0881-2

**Published:** 2017-08-08

**Authors:** Feng Li, Yuanxiu Li, Liming Sun, Xiaofei Li, Changji Yin, Xingjuan An, Xiaoli Chen, Yao Tian, Hao Song

**Affiliations:** 1 Key Laboratory of Systems Bioengineering (Ministry of Education), School of Chemical Engineering and Technology, Tianjin University, Tianjin, 300072 China; 2 SynBio Research Platform, Collaborative Innovation Centre of Chemical Science and Engineering, Tianjin University, Tianjin, 300072 China; 3 Petrochemical Research Institute, PetroChina Company Limited, Beijing, 102206 People’s Republic of China

**Keywords:** Microbial fuel cell, Synthetic biology, Xylose, *Shewanella oneidensis* MR-1

## Abstract

**Background:**

The microbial fuel cell (MFC) is a green and sustainable technology for electricity energy harvest from biomass, in which exoelectrogens use metabolism and extracellular electron transfer pathways for the conversion of chemical energy into electricity. However, *Shewanella oneidensis* MR-1, one of the most well-known exoelectrogens, could not use xylose (a key pentose derived from hydrolysis of lignocellulosic biomass) for cell growth and power generation, which limited greatly its practical applications.

**Results:**

Herein, to enable *S. oneidensis* to directly utilize xylose as the sole carbon source for bioelectricity production in MFCs, we used synthetic biology strategies to successfully construct four genetically engineered *S. oneidensis* (namely XE, GE, XS, and GS) by assembling one of the xylose transporters (from *Candida intermedia* and *Clostridium acetobutylicum*) with one of intracellular xylose metabolic pathways (the isomerase pathway from *Escherichia coli* and the oxidoreductase pathway from *Scheffersomyces stipites*), respectively. We found that among these engineered *S. oneidensis* strains, the strain GS (i.e. harbouring *Gxf1* gene encoding the xylose facilitator from *C. intermedi*, and *XYL1*, *XYL2*, and *XKS1* genes encoding the xylose oxidoreductase pathway from *S. stipites*) was able to generate the highest power density, enabling a maximum electricity power density of 2.1 ± 0.1 mW/m^2^.

**Conclusion:**

To the best of our knowledge, this was the first report on the rationally designed *Shewanella* that could use xylose as the sole carbon source and electron donor to produce electricity. The synthetic biology strategies developed in this study could be further extended to rationally engineer other exoelectrogens for lignocellulosic biomass utilization to generate electricity power.

**Electronic supplementary material:**

The online version of this article (doi:10.1186/s13068-017-0881-2) contains supplementary material, which is available to authorized users.

## Background

Bio-electrochemical systems enabled many practical applications in environments and energy fields [1–7], including microbial fuel cell (MFC) for simultaneous organic wastes treatment and electricity harvest [8–12], microbial electrolysis cells for hydrogen production [13–16], and microbial electrosynthesis for production of valuable chemicals from CO_2_ bioreduction [17–22]. Many mono-, di-saccharides as well as complex carbohydrates like starch and organics in wastewater and marine sediment have been used in MFCs for the production of electricity [8, 23, 24]. Xylose, one of primary ingredients from hydrolysis of lignocellulosic biomass, is the second most abundant carbohydrate after glucose in nature [25–27]. Conversion of xylose to electricity energy using MFC would thus provide a sustainable and green energy, which received increased attention in recent few years [24, 28–30]. However, xylose is hard to be effectively utilized by many microorganisms due to slow utilization rate and inefficient metabolic pathways of xylose [26, 31–35].

*Shewanella oneidensis*, one of the most well established metal-reducing exoelectrogens [36, 37], is capable of conducting extracellular electrons transfer (EET) through its metal-reducing (Mtr) pathway [38–42], being extensively studied for the optimization of MFC performance [40, 41, 43–47], MFC-based logic gate [48–50], bioremediation of toxic metals [51], etc., in recent decade. However, the wild-type (WT) *S. oneidensis* could only use three- (or two-) carbon substrates (e.g. lactate, pyruvate and acetate) as their carbon and energy sources, with an exception of N-acetyl-glucosamine (NAG) as a high-carbon carbohydrate [45, 52, 53], while common pentoses or hexoses (e.g. xylose and glucose), the most abundant composition of biomass, could not be utilized by the WT *S. oneidensis* owing to its incomplete sugar utilization pathways [36, 54, 55]. Such defect enormously restricted the wide applications of *S. oneidensis*.

Recently, several strategies were developed to use xylose for electricity generation in *Shewanella*-inoculated MFCs. Firstly, an adaptive evolution approach was developed to activate an otherwise silent xylose metabolic pathway, i.e. oxidoreductase pathway in the WT *S. oneidensis*, thus generating a *S. oneidensis* mutant XM1 that could metabolize xylose as the sole carbon and energy source [56]. Secondly, microbial consortia including fermenters and exoelectrogens were developed to accomplish xylose-powered MFCs, in which the engineered *Escherichia coli* played as a fermenter to metabolize xylose for the synthesis of metabolites such as lactate and formate to feed the *S. oneidensis* as the carbon source and electron donor, thus enabling an indirect utilization of xylose by *S. oneidensis* for bioelectricity production [24].

**Fig. 1:**
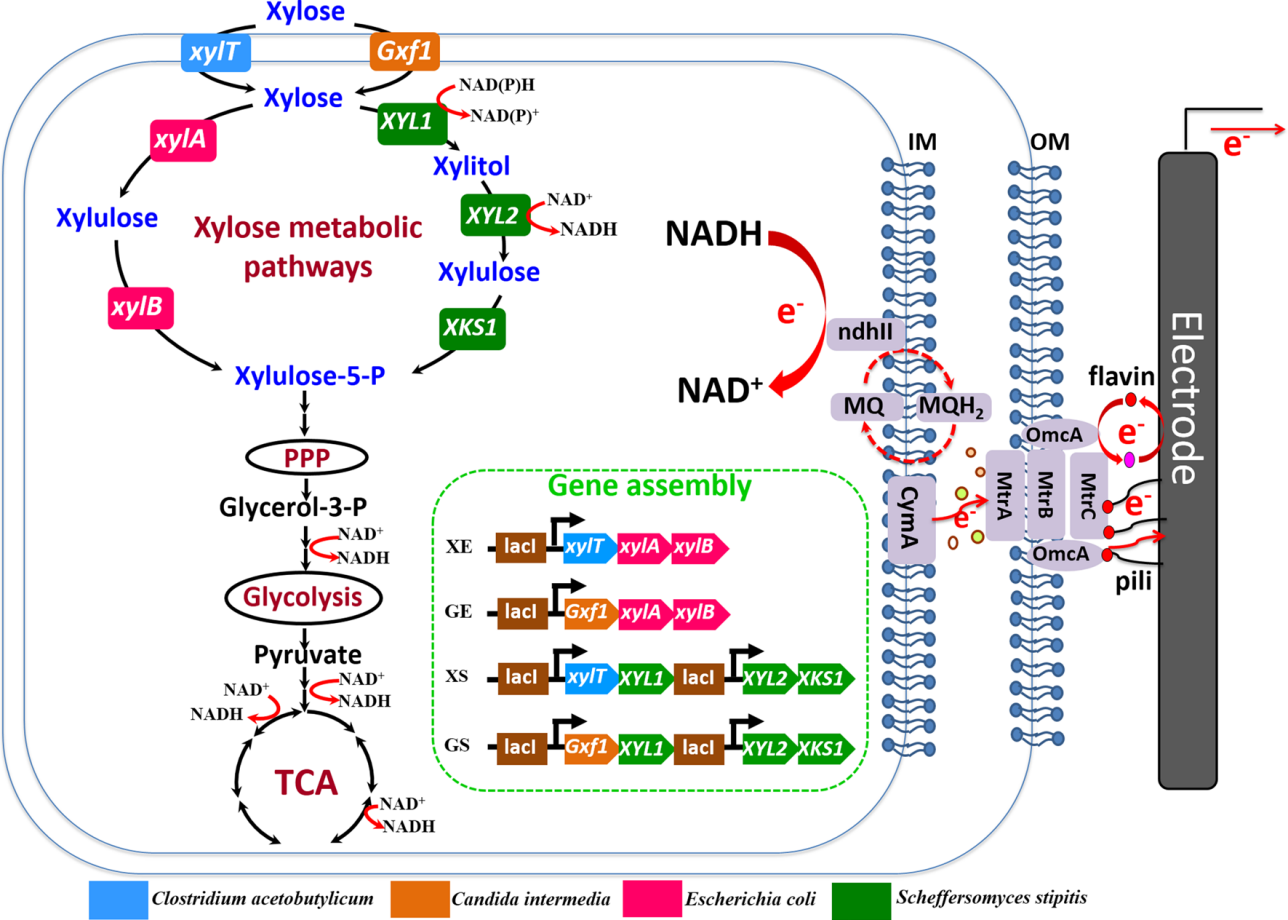
Synthetic biology strategies for the construction of four recombinant *S. oneidensis* strains (namely XE, GE, XS, and GS) to enable xylose utilization and electricity generation of *S. oneidensis*. Xylose transporter genes included *xylT* (the gene encoding d-xylose-proton symporter) from *Clostridium acetobutylicum* and *Gxf1* (the gene encoding glucose/xylose facilitator 1) from *Candida intermedia*. The xylose isomerase pathway included *xylA* (the gene encoding xylose isomerase) and *xylB* (the gene encoding xylulokinase) from *E. coli*. The oxidoreductase pathway included *XYL1* (the gene encoding d-xylose reductase), *XYL2* (the gene encoding xylitol dehydrogenase), and *XKS1* (the gene encoding d-xylulokinase) from *Scheffersomyces stipites*. Four gene assemblies (plasmids), namely XE, GE, XS, and GS (as shown in the *green-dash square*) were synthesized for the enhanced xylose transport and metabolism, which transformed into *S. oneidensis*, respectively, to construct four recombinant *S. oneidensis* strains

Herein, we used synthetic biology strategy to rationally engineer *S. oneidensis* that could use xylose as the sole carbon source and electron donor for electricity generation in MFCs. To enable *S. oneidensis* to be able to use xylose, the xylose transporters (i.e. glucose/xylose facilitator encoded by gene *Gxf1* from *Candida intermedia* [57, 58] and d-xylose-proton symporter encoded by gene *xylT* from *Clostridium acetobutylicum* [59]), synthetic isomerase pathway (including the genes *xylA* and *xylB* from *E. coli* [60]), and oxidoreductase (including from *Scheffersomyces stipites* [61]) pathway for xylose metabolism were heterologously expressed in *S. oneidensis* in a combinatorial way. Thus, four recombinant *S. oneidensis* strains were synthesized (see Fig. 1). Xylose-fed MFCs experiments proved that these engineered *S. oneidensis* MR-1 strains were conferred with the ability of utilizing xylose to produce electricity, and the engineered *S. oneidensis* strain GS provided the highest electricity generation. Compared with the *S. oneidensis* strain XM1 previously evolved by an adaptive evolution strategy [56], our rationally engineered *S. oneidensis* strains GS and XS (bearing the oxidoreductase pathway from *S. stipites*) showed a higher xylose consumption rate and a superior growth rate. In addition, the relative higher electricity generation by the GS strain than other engineered strains can be attributed to the higher intracellular riboflavin level and reducing equivalents in the GS. To the best of our knowledge, this was the first report on the rationally designed *Shewanella* that gained the expanded metabolic capability of using xylose as sole carbon source and electron donor to produce electricity.

## Results and discussion

### Engineered xylose-utilizing *S. oneidensis* strain via synthetic biology strategies

A few xylose metabolic pathways in microorganisms were found, including the oxidoreductase, isomerase, and Weimberg–Dahms pathways [56]. For example, *E. coli* is a robust and well-studied xylose scavenger [56, 62], which could metabolize xylose by the isomerase pathway; however, *S. stipites* [56, 61] could utilize oxidoreductase pathway for the metabolism of xylose. In the xylose isomerase pathway of *E. coli* [56, 60, 63], xylose isomerase encoded by the gene *xylA* converts xylose to xylulose, which is then phosphorylated by xylulokinase encoded by the gene *xylB* to xylulose 5-phosphate (X-5-P), and then enters the pentose phosphate pathway (see Fig. 1). In the oxidoreductase pathway of *S. stipites* [26], NAD(P)H-dependent xylose reductase encoded by the gene *XYL1* converts intracellular xylose to xylitol, which is then oxidized to xylulose by xylitol dehydrogenase (XDH) encoded by the gene *XYL2*. Xylulose is then phosphorylated by xylulokinase encoded by the gene *XKS1* to xylulose 5-phosphate (X-5-P), which enters the pentose phosphate pathway, similar to the isomerase pathway (see Fig. 1).

To facilitate convenient and fast multigene assembly in *S. oneidensis*, a Biobrick compatible vector named pYYDT including an IPTG-inducible promoter PlacIq-lacIq-Ptac was well developed in our laboratory (Additional file 1: Figure S1B) [64]. Furthermore, to avoid the codon usage bias and prevent blocked translation due to shortage of tRNAs for rare codons between *S. oneidensis* and other bacteria, in vitro chemical synthesis of codon-optimized genes instead of direct cloning from other bacteria was used. The xylose metabolic pathway was then assembled by several routines of Biobrick ligation steps of the relevant genes. With the combinations of the two xylose transporters and the two xylose-utilizing metabolic pathways (the isomerase and the oxidoreductase pathways), four recombinant *S. oneidensis* strains harbouring engineered gene assembly (plasmid) for enhanced xylose transport and metabolism were synthesized, respectively, which were XE (including the gene *xylT* for xylose symporter, *xylA* and *xylB* for the xylose isomerase pathway), GE (including *Gxf1* for the xylose facilitator, *xylA* and *xylB* for the xylose isomerase pathway), XS (including *xylT* for xylose symporter, and *XYL1, XYL2,* and *XKS1* for the xylose oxidoreductase pathway), and GS (including *Gxf1* for the xylose facilitator, and *XYL1, XYL2,* and *XKS1* for the xylose oxidoreductase pathway).

### Evaluation of xylose utilization and cell growth of the recombinant *S. oneidensis*

The cell growth and xylose consumption by the wild-type (WT, harbouring the pYYDT empty vector) and four genetically engineered *S. oneidensis* strains (i.e. harbouring XE, GE, XS, and GS, respectively) were evaluated in SBM supplemented with 5 mM xylose as the sole carbon source.

**Fig. 2:**
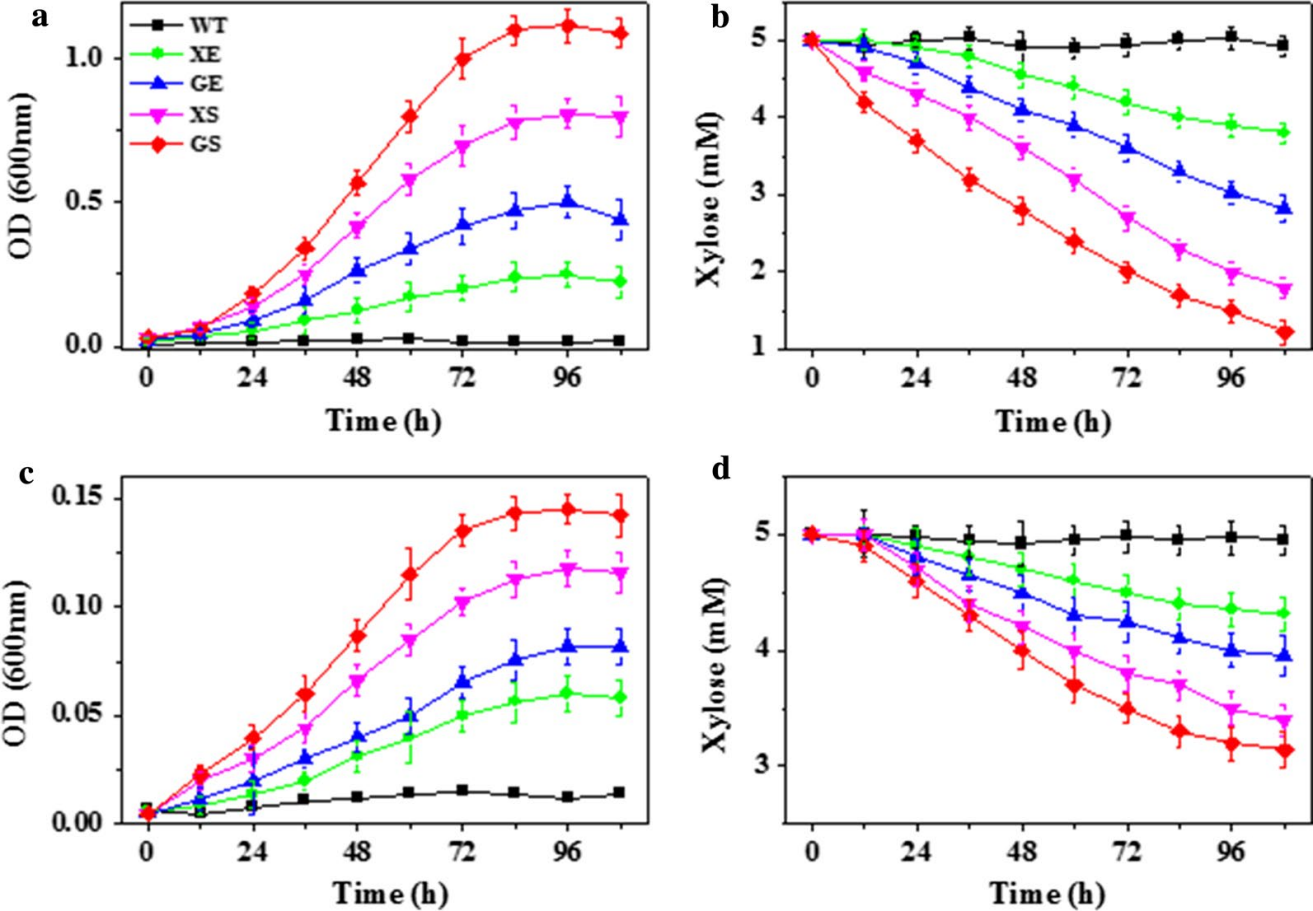
Growth curves and xylose consumption of the WT and the recombinant *S. oneidensis* strains. **a** Aerobic growth curve (OD_600_ ~ t) in SBM supplemented with 5 mM xylose. **b** Xylose consumption under aerobic conditions. **c** Anaerobic growth curve (OD_600_ ~ t) in SBM supplemented with 5 mM xylose. **d** Xylose consumption under anaerobic conditions. The *error bars* were calculated from triplicate experiments

Under aerobic conditions, the WT *S. oneidensis* strain showed almost no growth and xylose consumption, while the four engineered *S. oneidensis* strains showed a superior growth over the WT *S. oneidensis* strain. In addition, the growth rate of the engineered strains XS and GS (harbouring the oxidoreductase pathway) was faster than that of the strains XE and GE (harbouring the isomerase pathway) (Fig. 2a). The engineered strain XS and GS consumed xylose at a rate of ~28.1 and ~35.2 μM/h, which was faster than that of the engineered strains XE and GE (~11.2 and ~20.3 μM/h) (Fig. 2b). Thus, the rate of xylose consumption of these engineered strains was in good agreement with that of the growth rate, respectively.

The anaerobic respiratory capabilities of the WT and the recombinant *S. oneidensis* were also determined under anaerobic conditions with xylose as the sole electron donor and fumarate as the electron acceptor. Similar to the aerobic conditions, the four genetically engineered strains grew faster than that of the WT strain. The recombinant strains XS and GS (harbouring the oxidoreductase pathway) consumed xylose at a rate of ~14.8, and ~17.2 μM/h, respectively, which had a faster xylose consumption rate than that of the strains XE and GE (harbouring the isomerase pathway, ~6.3 and ~9.7 μM/h, respectively) (Fig. 2c, d). Furthermore, the engineered strain GS could intake xylose faster than XS, which indicated that the glucose/xylose facilitator Gxf1 enabled a higher xylose transportation than that of the D-xylose-proton symporter XylT. It was revealed that sugar uptake via facilitated diffusion by Gxf1 required less energy (ATP) than proton symport XylT, and thus the facilitator protein would probably be more efficient with higher substrate affinity under oxygen-limited or anaerobic conditions where ATP production is restricted in our MFC conditions [58, 65]. In addition, all recombinant *S. oneidensis* strains were able to utilize lactate as the sole carbon source at a rate similar to that of the WT strain, suggesting that the lactate metabolism of *S. oneidensis* was not altered by such engineering efforts (data not shown).

Thus, our results indicated that the introduction of one of the synthetic xylose transporters (the d-xylose-proton symporter from *C. acetobutylicum* and the glucose/xylose facilitator from *C. intermedia*) and one of the metabolic pathways (i.e. the isomerase pathway from *E. coli* and the oxidoreductase pathways from *S. stipites*) could successfully confer *Shewanella* strains with the ability of utilizing xylose as the sole carbon source for the cell growth. Especially, our rationally designed *S. oneidensis* strains XS and GS (bearing the oxidoreductase pathway from *S. stipites*) showed a higher consumption of xylose and a superior growth rate than that of the *S. oneidensis* strain XM1 (that was recently developed through an adaptive evolution strategy) [56]. *Escherichia coli* (the BL21 strain) harbouring those genes related to xylose transport and metabolism exhibited a superior xylose consumption rate (~455 μM/h), i.e. ~12 times faster than that of the engineered *S. oneidensis* GS (~35.2 μM/h) (Additional file 1: Figure S2). This result indicated that although the engineered *S. oneidensis* was enabled the capability of xylose utilization, there was much room to further improve its xylose consumption rate by synthetic biology endeavours.

### MFC performance and bio-electrochemical analyses

MFC was used to examine the extracellular electron transfer and power generation by the engineered *S. oneidensis* MR-1 using xylose as the sole carbon source. The WT and the engineered *S. oneidensis* strains were inoculated into the anodic chamber of MFCs, respectively, with a 2 kΩ external resistor, across which the voltage output was recorded.

**Fig. 3:**
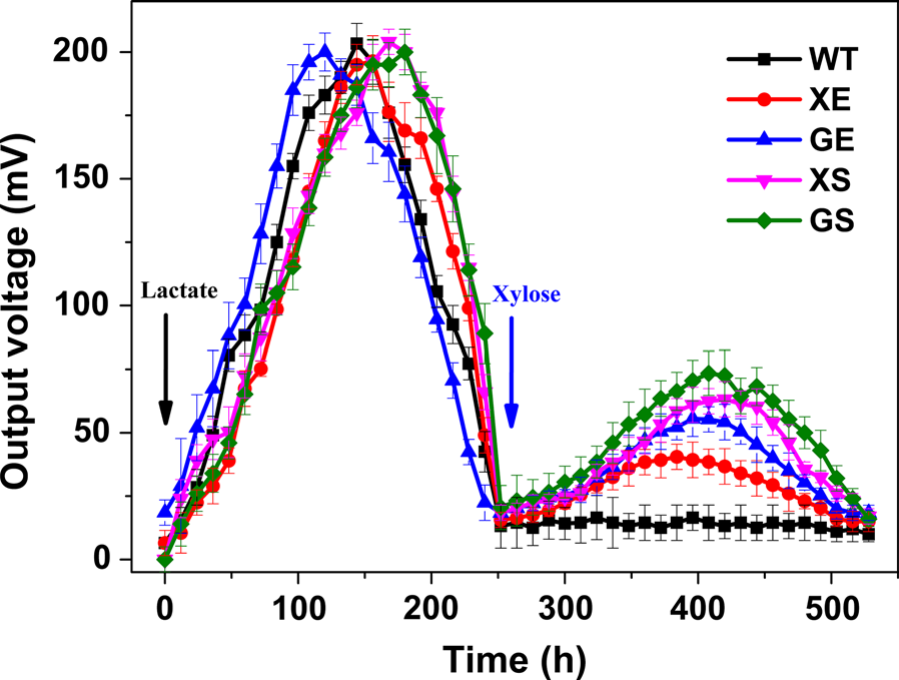
Output voltage of the WT and four recombinant *S. oneidensis* strains (XE, GE, XS, and GS) with different carbon sources in the anodic chamber of MFCs. The output voltage of the four strains XE, GE, XS, and GS, using lactate and xylose as the carbon source, respectively. 18 mM lactate (the favourable carbon source of *Shewanella*) was added at the initiation of MFC operations (as indicated by the *black arrow*). Upon the depletion of lactate and vanishing of electricity output, 18 mM xylose was added at ~260 h as the carbon source (as indicated by the *blue arrow*) to illustrate the power generation capability of these *S. oneidensis* strains using xylose as the sole carbon source. The *error bars* were calculated from triplicate experiments

**Fig. 4:**
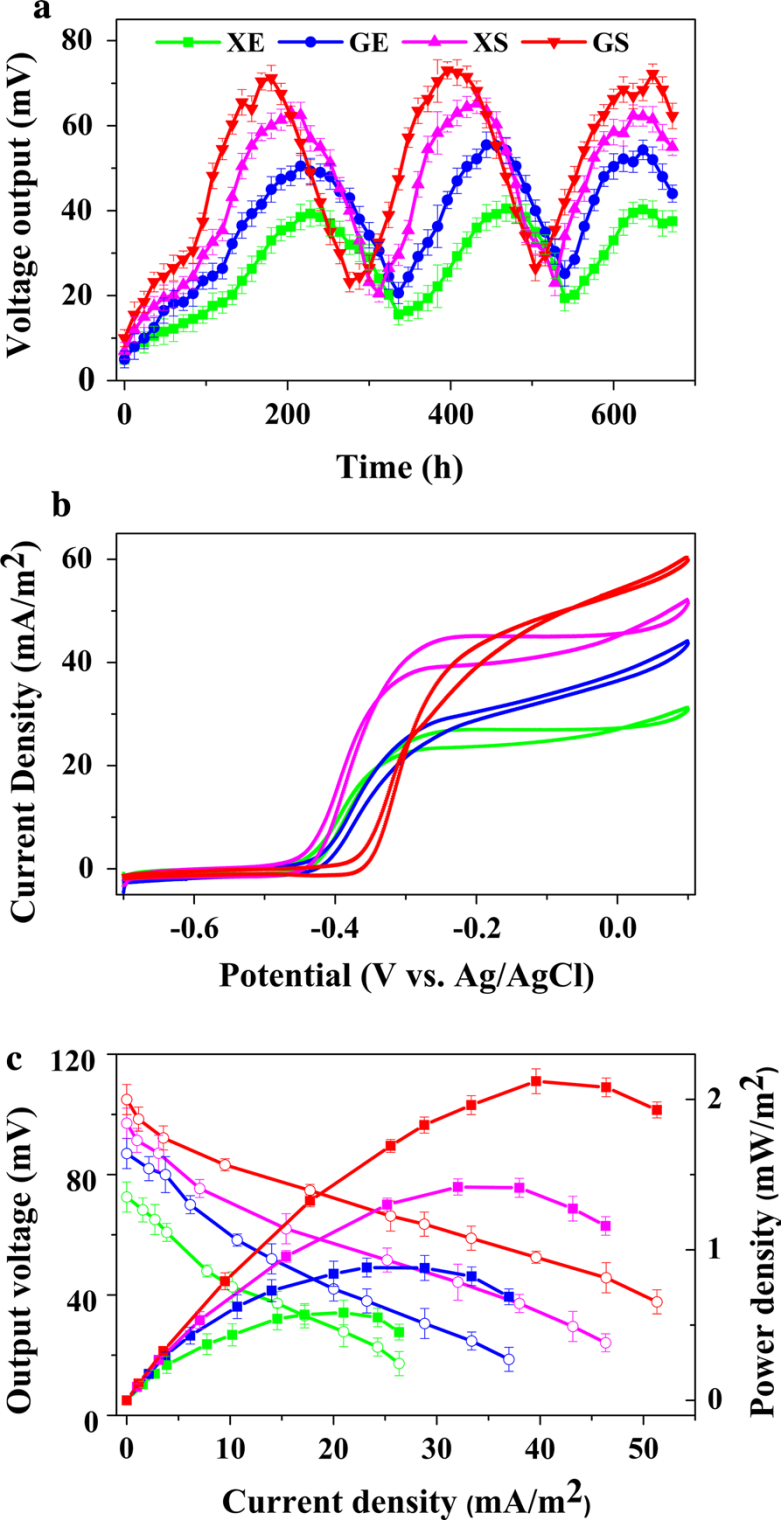
Bio-electrochemical characterization of the extracellular electron transfer (EET) and electricity output capability of the four engineered *S. oneidensis* strains (namely XE, GE, XS, and GS) with xylose as the sole carbon source. **a** Voltage output of the four engineered *S. oneidensis* strains (XE, GE, XS, and GS) in the semi-batch xylose-fed multiple operational cycles of MFCs. Upon vanishing of electricity output and depletion of xylose at each MFC cycle, 18 mM xylose was added to maintain the multiple MFC operation cycles. **b** Cyclic voltammetry (CV) at the scan rate of 1 mV/s.** c** Polarization curves and power density output curves of the MFCs inoculated with the four recombinant *S. oneidensis* strains, respectively. The *error bars* were calculated from triplicate experiments

Initially, 18 mM lactate was used (as the favourable carbon source of *Shewanella*) to feed the engineered *S. oneidensis* strains in MFCs to verify the capacity of power output of each strain (Fig. 3). After the output voltage decreased to baseline levels (indicating the depletion of lactate), 18 mM xylose was added into the anodic chamber as the carbon source. Obviously, the output voltages of these engineered *S. oneidensis* strains with xylose as the carbon source were lower than those of lactate as the carbon source, because lactate is the favourable carbon source for the growth and respiration of *Shewanella* (Fig. 3). When lactate was used as the carbon source, the maximum output voltages could increase to ~205 ± 7.2 mV (*n* = 3) for both the WT and engineered *S. oneidensis* strains. However, the WT *S. oneidensis* strain could barely generate any voltage output when xylose was used as the carbon source, which indicated that the WT *S. oneidensis* could not utilize xylose. Upon genetic programming of the xylose transporter and metabolic pathway into *S. oneidensis*, the recombinant *S. oneidensis* strains, namely XE, GE, XS, and GS, could generate a maximum output voltage of ~40.5 ± 5.1, ~55.5 ± 4.8, ~63.2 ± 6.2, and ~73.4 ± 5.8 mV (*n* = 3), respectively. Furthermore, the multiple cycles of voltage output of these genetically engineered *S. oneidensis* strains showed the stability of power generation in the semi-batch xylose-fed MFCs (Fig. 4a).

We observed that the strains GS and XS harbouring the synthetic oxidoreductase xylose metabolic pathway could generate a higher voltage output than those of the XE and GE harbouring the synthetic isomerase xylose metabolic pathway (Fig. 4a). Bio-electrochemical analyses were further conducted to study the EET efficiency of these rationally engineered strains in MFCs. The cyclic voltammetry (CV) at 1 mV/s was applied to reveal the redox reaction kinetics at the interfaces of bacterial cells and anodes. As shown in Fig. 4b, there were typical redox peaks of flavins in the CV curves starting from around −0.4 V (vs. Ag/AgCl), which showed that flavins-mediated extracellular electron transfer was the dominating mechanism for bioelectricity production in these strains [64, 66]. The power output curves (output voltage vs. current density) and the polarization curves (power density vs. current density), which were obtained by varying load resistances to show the dependence of voltage and power on the current, helped to further investigate the bioelectricity generation capability of the engineered *S. oneidensis* strains (Fig. 4c). Notably, the dropping slope of the polarization curve obtained from the engineered *S. oneidensis* strain GS (harbouring the xylose facilitator and the xylose oxidoreductase pathway) was smaller than those obtained from the other three engineered *S. oneidensis* stains (i.e. XE, GE, XS), implying that the internal charge transfer resistance of the MFC inoculated with GS was relatively smaller (Fig. 4c). The power density were calculated, which showed that the engineered *S. oneidensis* strain GS obtained a maximum power density of ~2.1 ± 0.1 mW/m^2^ (*n* = 3), which was ~0.3, ~0.9, ~1.1 times higher than that of XE, GE, and XS, respectively (Fig. 4c). Previous xylose-fed MFCs generally used sludge, natural or synthetic microbial consortia, the power generation of which were in the range of 6.3–2330 mW/m^2^ (as shown in Additional file 2: Table S1), higher than that of our recombinant *S. oneidensis* strain. Thus, future engineering of *Shewanella oneidensis* to enable higher output electricity remained of paramount importance.

**Fig. 5:**
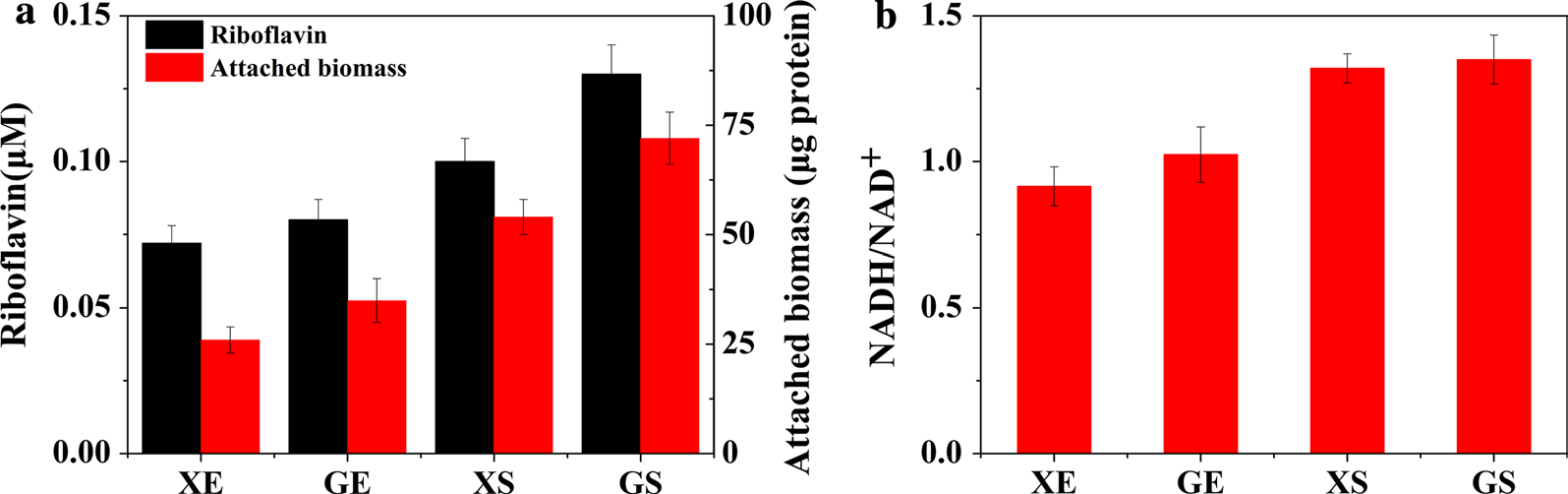
Biochemical analyses of the four engineered *S. oneidensis* strains harbouring either the synthetic isomerase pathway (XE and GE) or the oxidoreductase pathway (XS and GS), respectively. **a** Riboflavin concentration in the anolytes of MFCs, and the attached biomass of each strain on anode surfaces. **b** Quantitative measurements of the ratio of NADH/NAD^+^ in these engineered *S. oneidensis* strains in MFCs. All *error bars* were calculated from triplicate experiments

Biochemical characterizations showed that the engineered strains GS and XS had a higher utilization efficiency of xylose and higher growth rate, and a more efficient formation of biofilm attached on the anodes (Fig. 5a). Meanwhile, the engineered strains GS and XS could also generate higher intracellular reducing equivalents (i.e. NADH/NAD^+^, Fig. 5b). Such a high intracellular releasable electron pool (i.e. NADH) had resulted from the oxidative reaction of xylitol to xylulose, mediated by the reduction of NAD^+^ to NADH in the oxidoreductase pathway [65, 67–69]. Both the efficient biofilm formation on the anodes [24, 70] and higher intracellular reducing equivalents [71, 72] in the engineered *S. oneidensis* strains GS and XS synergistically enabled an enhanced EET efficiency and electricity generation. In addition, an increase in the secretion of riboflavin in the recombinant strains also enabled an increase in the output voltage of MFCs. The increased biosynthesis of riboflavin would be attributed to the biosynthesis of xylulose 5-phosphate (X-5-P) owing to the heterologously introduced xylose metabolism pathway (i.e. the oxidoreductase pathway). X-5-P, as a metabolic product of the oxidoreductase pathway, was converted to ribulose-5-P, a crucial precursor for the biosynthesis of riboflavin, by ribulose-phosphate 3-epimerase encoded by the *rpe* gene in the pentose phosphate pathway. Subsequently, ribulose-5-P and guanosine triphosphate (GTP) were converted to riboflavin via the riboflavin biosynthesis pathway (Additional file 1: Figure S3) [24, 36, 53].

## Conclusions

To the best of our knowledge, this research is the first to use synthetic biology strategy to rationally engineer *S. oneidensis* MR-1 to enable direct utilization of xylose as the sole carbon source and electron donor for bioelectricity production in MFCs. The efficient xylose metabolic pathways (the isomerase pathway or the oxidoreductase pathway) combined with two different xylose transporters were heterologously expressed in *S. oneidensis* MR-1 to construct four engineered *S. oneidensis* strains (namely XE, GE, XS, and GS), which could successfully utilize xylose under anaerobic and aerobic conditions. These recombinant *S. oneidensis* strains could generate bioelectricity in MFCs with xylose as the sole carbon source and electron donor. The maximum power density of the MFC inoculated with the engineered *S. oneidensis* strain GS (harbouring the xylose facilitator and the xylose oxidoreductase pathway) could reach ~2.1 ± 0.1 mW/m^2^. This rationally engineered xylose transport and metabolic pathway significantly expanded the spectrum of carbon source that could be used by *S. oneidensis*. In the foreseeable future, with continuous development of synthetic biology strategies [73–75] to engineer exoelectrogens, a diverse array of organics such as lignocellulosic biomass and recalcitrant wastes may be more efficiently converted to electricity power.

## Methods

### In vitro gene synthesis

The information and coding sequences of the genes (Additional file 2: Tables S2 and S3) were extracted from the NCBI database and adapted for optimal expression in *S. oneidensis* MR-1 by a Java codon adaption tool (JCAT) in order to prevent blocked translation due to shortage of tRNAs for rare codons [49]. Each gene component was synthesized as a Biobrick [76, 77], and restriction enzyme sites of *Eco*RI, *Xba*I, *Spe*I, and *Sb*fI were avoided in the codon-optimized sequences. The optimized gene sequence was flanked by an upstream prefix (containing *Eco*RI and *Xba*I), a RBS site (BBa_B0034, iGEM) located at 6 bp ahead of the start codon, and a downstream suffix (containing *Spe*I and *Sbf*I) (Additional file 1: Figure S1A). The designed gene sequences were synthesized in vitro, verified by Sanger sequencing (AuGCT, China).

### Plasmid construction, transformation, and culture conditions

All plasmid constructions were performed in *E. coli* Trans T1. The *E. coli* strains were cultured in the LB (Luria–Bertani) medium at 37 °C with 200 rpm. The plasmid to be transformed into *S. oneidensis* MR-1 (ATCC 700550) was firstly transformed into the plasmid donor strain *E. coli* WM3064 (auxotroph), and then transferred into *S. oneidensis* by conjugation. Then, 100 μg/ml 2, 6-diaminopimelic acid (DAP) was added for the growth of *E. coli* WM3064. Whenever needed, 50 μg/ml kanamycin was added in the culture medium for plasmid maintenance. All the strains and plasmids used in this study are listed in Table (Additional file 2: Table S4).

### Determination of cell growth and xylose utilization

To determine cell growth and xylose utilization under both aerobic and anaerobic conditions, 0.5 ml of the wild-type (WT) or engineered xylose-utilizing *S. oneidensis* strain culture suspension was inoculated into 15 ml *Shewanella* basal medium (SBM) [53] (Additional file 2: Table S5), supplemented with 5 mM xylose as the electron donor and carbon source in the test tube. When needed, 10 mM sodium fumarate was supplemented as the electron acceptor, which was stoichiometrically sufficient from both theoretical calculations and experimental validations (Additional file 1: Figure S4). The cell cultures were incubated at 30 °C, and samples were withdrawn periodically for the determination of cell density (optical density at 600 nm, i.e. OD_600_) and xylose consumption. The OD_600_ was measured by an ultraviolet and visible spectrophotometer (TU-1810, Beijing, China).

### BES setup

To evaluate the efficiency of extracellular electron transfer (EET), the overnight *Shewanella* culture suspension (1.5 ml) was inoculated into 150 ml fresh LB broth at 30 °C with shaking (200 rpm) till the OD_600_ reached 0.6–0.8. Then, the cells were harvested by centrifugation and washed 3 times with fresh M9 buffer (Additional file 2: Table S6). The cell pellets were subsequently re-suspended in 140 ml electrolyte (5% LB broth plus 95% M9 buffer supplemented with 18 mM lactate or xylose). 50 μg/ml kanamycin was added to ensure consistent culture condition. The medium was supplemented with 0.1 mM IPTG as the inducer of the tac promoter. Our previous experiments proved that IPTG had no effect on the cell physiology and EET of *Shewanella* [70]. The dual-chamber MFCs were used in this study, namely the anodic and cathodic chambers (140 ml working volume) separated by the nafion 117 membrane (DuPont Inc., USA), were the same as those used in the previous study. Carbon cloth was used as the electrodes for both the anode (2.5 cm × 2.5 cm, i.e. the geometric area is 6.25 cm^2^) and the cathode (2.5 cm × 3 cm). The cathodic electrolyte consisted of 50 mM K_3_[Fe(CN)_6_] in 50 mM K_2_HPO_4_ and 50 mM KH_2_PO_4_ solution. To measure the voltage generation, a 2 kΩ external resistor was connected into the external circuit of MFCs, and the output voltage (*V*) across the external loading resistor (*R*) was measured by a digital multimeter (DT9205A).

### Electrochemical analyses

Cyclic voltammetry (CV) was performed in a three-electrode configuration with an Ag/AgCl reference electrode on a CHI 1000C multichannel potentiostat (CH Instrument, Shanghai, China). At the pseudo-steady state of MFCs, the polarization curves were obtained by varying the external resistor. Current density (*I*) was calculated as *I* = *V* (output voltage)/*R* (external resistance), and power density (*P*) was calculated as *P* = *V* × *I*. Then, the *I* and *P* were normalized to the projected geometric area of the anode to obtain the current density and power density, respectively [78].

### Quantification of metabolites

For the quantification of riboflavin, the samples in the MFC supernatant were firstly centrifuged (35,000 rpm for 20 min) and filtered (0.22 µm), and then, the eluted media were detected by a liquid chromatograph-tandem mass spectrometer (LC–MS) (Agilent LCMS-1290-6460) in a positive ion mode using a Waters XBridge C8 column (2.1 × 100 mm; particle size: 3.5 µm). Xylose in the anolytes were analysed using a high-performance liquid chromatography (HPLC) system equipped with a diode array detector. Sulphuric acid (5 mM) was used as the mobile phase flowing at 0.6 ml/min through the Aminex HPX-87H column (Bio-Rad, USA), which was incubated at 50 °C. Signals at 190 nm were used to quantify xylose.

### Quantification of intracellular NADH/NAD^+^

Cells (10 ml) were collected by centrifugation (10,000 rpm at 4 °C for 5 min) and immediately re-suspended in 300 μl of 0.2 M HCl (for NAD^+^) or 0.2 M NaOH (for NADH). The suspensions were boiled for 7 min, rapidly quenched in an ice bath, and added with 300 μl of 0.1 M NaOH (for NAD^+^) or 0.1 M HCl (for NADH). Cell debris was removed by centrifugation at 10,000 rpm for 10 min, and the supernatant was used in a cycling assay to determine the amounts of NAD^+^ and NADH [79, 80]. Meanwhile, the cell concentration for the detection of NAD^+^ and NADH concentration was detected by plate counts on LB agar.

### Measurement of electrode-attached biomass

The electrode was placed in a 50-ml tube containing 5 ml of 0.2 mol/l NaOH, then vortexed for 2 min, and incubated in a water bath to lyse cells at 96 °C for 30 min. The extracts were tested by bicinchoninic acid protein assay kit (Solarbio, China) after being cooled to room temperature.

## Additional files

**Additional file 1: Figure S1.** Construction of synthetic xylose metabolic pathways in *Shewanella oneidensis* MR-1. (A) Schematic of the plasmid with a synthesized functional fragment of genes. The restriction sites EcoRI and XbaI with the ribosome binding site (RBS) are located upstream of each codon-optimized gene sequence, while the restrictions SpeI and PstI are located downstream of the gene. (B) Four plasmid constructs with xylose utilization pathways. To construct the multigene assembly in *S. oneidensis*, a Biobrick compatible expression vector pYYDT was adopted, which was previously constructed in our laboratory. Layout of the four plasmid constructs containing gene components in the xylose pathway examined in this study. **Figure S2**. Xylose consumption rate by *E. coil* (BL21) and by the recombinant *S. oneidensis* strain. The error bars were calculated from triplicate experiments. **Figure S3**. Metabolic pathway of riboflavin synthesis from xylose fermentation in *S. oneidensis*. A synthetic intracellular xylose metabolic pathway, i.e. the oxidoreductase pathway including genes *XYL1*, *XYL2* and *XKS1* from *S. stipites*, is incorporated into *S. oneidensis* MR-1 to enable the direct utilization of xylose. Xylulose 5-phosphate, as a metabolite in the oxidoreductase pathway, was converted to ribulose-5-P by ribulose-phosphate 3-epimerase (encoded by the *rpe* gene) in the pentose phosphate pathway, which was a crucial precursor for the biosynthesis of riboflavin via the riboflavin synthesis pathway. **Figure S4.** Xylose consumption under anaerobic conditions with 10 mM and 50 mM fumarate. The error bars were calculated from triplicate experiments.

**Additional file 2: Table S1.** Summary of the reported energy output of Xylose-Fed MFCs. **Table S2.** Genes used in this study. **Table S3.** Synthesized sequences of genes in this study. **Table S4.** Strains and plasmids used in this study. **Table S5.** Main constituents for *S. oneidensis* basal medium (SBM). **Table S6.** Main constituents for M9 buffer.

## Abbreviations

EET: extracellular electron transfer
MQ: methyl naphthoquinone
IM: inner membrane
OM: outer membrane
CymA: inner membrane tetraheme *c*-type cytochromes
Mtr: metal-reducing
MtrA: periplasmic decaheme
MtrB: β-barrel trans-OM protein
MtrC and OmcA: two OM decaheme *c*-type cytochromes
TCA: tricarboxylic acid cycle
ndhII: NADH dehydrogenase
P: phosphate
PEP: phosphoenolpyruvate
NAD^+^: nicotinamide adenine dinucleotide
NADH: reduced nicotinamide adenine dinucleotide
NADP^+^: nicotinamide adenine dinucleotide phosphate
NADPH: reduced nicotinamide adenine dinucleotide phosphate
ATP: adenosine triphosphate
ADP: adenosine diphosphate
X-5-P: xylulose 5-phosphate
GTP: guanosine triphosphate
IPTG: isopropyl β-D-1-thiogalactopyranoside
DAP: 2, 6-diaminopimelic acid
MFC: microbial fuel cell
OD: optical density
CV: cyclic voltammetry
XDH: xylitol dehydrogenase
NAG: N-acetyl-glucosamine
