# Supplementary material for: Engineering Shewanella oneidensis enables xylose-fed microbial fuel cell
Source: Biotechnol Biofuels. 2017 Aug 8;10:196. doi: 10.1186/s13068-017-0881-2 (PMC5549365; doi:10.1186/s13068-017-0881-2)
Supplement: Supplementary file 1 — Additional file 1: Figure S1. Construction of synthetic xylose metabolic pathways in Shewanella oneidensis MR-1. (A) Schematic of the plasmid with a synthesized functional fragment of genes. The restriction sites EcoRI and XbaI with the ribosome binding site (RBS) are located upstream of each codon-optimized gene sequence, while the restrictions SpeI and PstI are located downstream of the gene. (B) Four plasmid constructs with xylose utilization pathways. To construct the multigene assembly in S. oneidensis, a Biobrick compatible expression vector pYYDT was adopted, which was previously constructed in our laboratory. Layout of the four plasmid constructs containing gene components in the xylose pathway examined in this study. Figure S2. Xylose consumption rate by E. coil (BL21) and by the recombinant S. oneidensis strain. The error bars were calculated from triplicate experiments. Figure S3. Metabolic pathway of riboflavin synthesis from xylose fermentation in S. oneidensis. A synthetic intracellular xylose metabolic pathway, i.e. the oxidoreductase pathway including genes XYL1, XYL2 and XKS1 from S. stipites, is incorporated into S. oneidensis MR-1 to enable the direct utilization of xylose. Xylulose 5-phosphate, as a metabolite in the oxidoreductase pathway, was converted to ribulose-5-P by ribulose-phosphate 3-epimerase (encoded by the rpe gene) in the pentose phosphate pathway, which was a crucial precursor for the biosynthesis of riboflavin via the riboflavin synthesis pathway. Figure S4. Xylose consumption under anaerobic conditions with 10 mM and 50 mM fumarate. The error bars were calculated from triplicate experiments. [file 13068_2017_881_MOESM1_ESM.docx]

**Supporting figures**

**
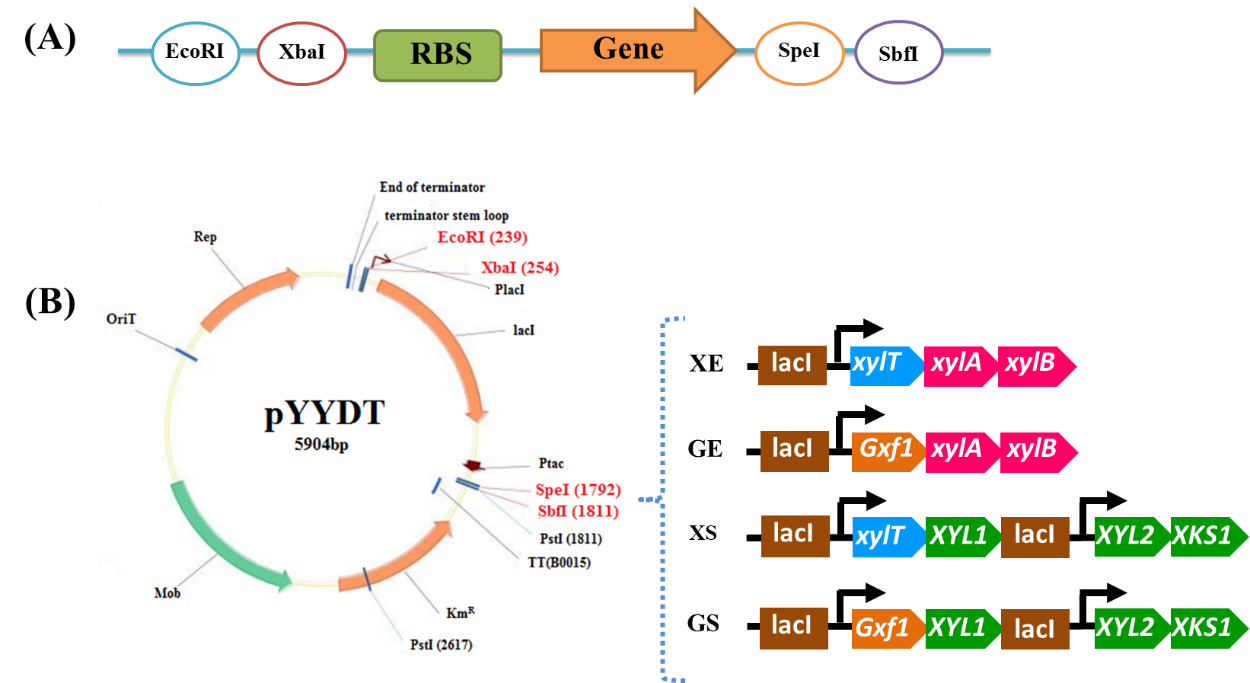
**

**Figure S1.** Construction of synthetic xylose metabolic pathways in *Shewanella oneidensis* MR-1. (A) Schematic of the plasmid with a synthesized functional fragment of genes. The restriction sites EcoRI and XbaI with the ribosome binding site (RBS) are located upstream of each codon-optimized gene sequence, while the restrictions SpeI and PstI are located downstream of the gene[[1](#_ENREF_1)]. (B) Four plasmid constructs with xylose utilization pathways. To construct the multi-gene assembly in *S. oneidensis*, a Biobrick compatible expression vector pYYDT was adopted, which was previously constructed in our laboratory[[2](#_ENREF_2), [3](#_ENREF_3)]. Layout of the four plasmid constructs containing gene components in the xylose pathway examined in this study.

**Figure S2.** Xylose consumption rate by *Escherichia coil* (BL21) and by the recombinant *S. oneidensis* strain (GS). The error bars were calculated from triplicate experiments.

The *E. coil* strain BL 21 exhibited a superior xylose consumption rate (~455 μM/h), ~12 times faster than that of the engineered *S. oneidensis* GS (~35.2 μM/h). This result indicated that although the engineered *S. oneidensis* was enabled the capability of xylose utilization, there was still much room to further improve its xylose consumption rate by synthetic biology endeavors.

**
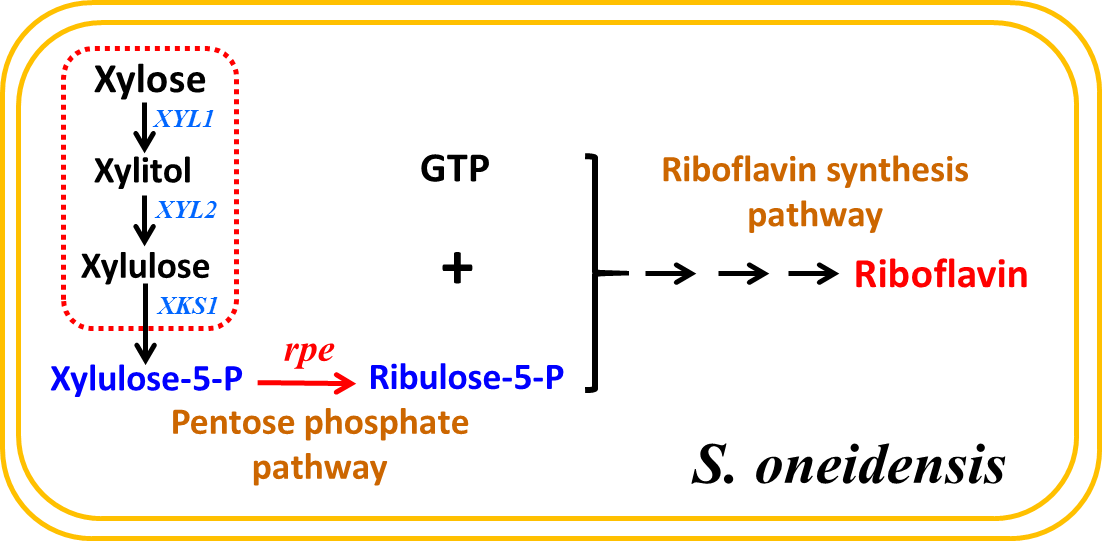
**

**Figure S3.** Metabolic pathway of riboflavin synthesis from xylose fermentation in *S. oneidensis*. A synthetic intracellular xylose metabolic pathway, i.e., the oxidoreductase pathway including genes *XYL1*, *XYL2* and *XKS1* from *Scheffersomyces stipites*, is incorporated into *S. oneidensis* MR-1 to enable the direct utilization of xylose. Xylulose 5-phosphate, as a metabolite in the oxidoreductase pathway, was converted to ribulose-5-P by ribulose-phosphate 3-epimerase (encoded by the *rpe* gene) in the pentose phosphate pathway, which was a crucial precursor for the biosynthesis of riboflavin via the riboflavin synthesis pathway[[4-6](#_ENREF_4)]. Abbreviations: GTP, guanosine triphosphate; P, phosphate.

**Figure S4.** Xylose consumption under anaerobic conditions with 10 mM and 50 mM fumarate. The error bars were calculated from triplicate experiments.

To verify that 10 mM fumarate was sufficient and would not limit the xylose consumption, we performed two new experiments. Firstly, we used HPLC to measure the level of fumarate in xylose consumption under anaerobic conditions. We found the residual fumarate level would be kept at 2.2 mM at the end of the experiments, which indicated the amount of fumarate is stoichiometrically sufficient in the xylose consumption experiments. Secondly, we conducted a few experiments of xylose consumption with a higher concentration of fumarate (50 mM) over the theoretical value (20 mM) with the engineered *S. oneidensis* GS. Our results dedicated that there was a negligible difference in the rate of xylose consumption between different levels of fumarate (the electron acceptor), as shown in Figure S4.

Furthermore, based on the stoichiometric calculation, 1 mol xylose could theoretically generate 4 mol electrons when xylose was oxidized to acetate (xylose metabolism via the phosphoketolase pathway generates equimolar amounts of lactate and acetate, while 1 mol lactate could generated 4 mol electrons when transformed to acetate), and 1 mol fumarate could accept 2 mol electrons upon being reduced to succinate. Thus, 10 mmol fumarate that we used in the experiments can accept 20 mmol electrons from xylose. However, 5 mmol xylose consumed by the engineered *Shewanella* would generate 20 mmol electrons under anaerobic conditions. So, the amount of fumarate (10 mM) used as the electron acceptor was stoichiometric sufficient when xylose (5 mM) was used as electron donor and carbon source.

**References**

1. Hu Y, Yang Y, Katz E, Song H: Programming the quorum sensing-based AND gate in *Shewanella oneidensis* for logic gated-microbial fuel cells. Chem Commun 2015, 51(20):4184-87.

2. Yang Y, Ding Y, Hu Y, Cao B, Rice SA, Kjelleberg S *et al*: Enhancing Bidirectional Electron Transfer of Shewanella oneidensis by a Synthetic Flavin Pathway. ACS Synth Biol 2015, 4(7):815-23.

3. Lin T, Bai X, Hu Y, Li B, Yuan Y, Song H *et al*: Synthetic Saccharomyces cerevisiae-Shewanella oneidensis consortium enables glucose-fed high-performance microbial fuel cell. AIChE Journal 2016.

4. Yang Y, Wu Y, Hu Y, Cao Y, Poh CL, Cao B *et al*: Engineering electrode-attached microbial consortia for high-performance xylose-fed microbial fuel cell. ACS Catal 2015, 5(11):6937-45.

5. Fredrickson JK, Romine MF, Beliaev AS, Auchtung JM, Driscoll ME, Gardner TS *et al*: Towards environmental systems biology of *Shewanella*. Nat Rev Microbiol 2008, 6(8):592-603.

6. Flynn CM, Hunt KA, Gralnick JA, Srienc F: Construction and elementary mode analysis of a metabolic model for Shewanella oneidensis MR-1. Biosystems 2012, 107(2):120-28.
