# Supplementary material for: Engineering Shewanella oneidensis enables xylose-fed microbial fuel cell
Source: Biotechnol Biofuels. 2017 Aug 8;10:196. doi: 10.1186/s13068-017-0881-2 (PMC5549365; doi:10.1186/s13068-017-0881-2)
Supplement: Supplementary file 2 — Additional file 2: Table S1. Summary of the reported energy output of Xylose-Fed MFCs. Table S2. Genes used in this study. Table S3. Synthesized sequences of genes in this study. Table S4. Strains and plasmids used in this study. Table S5. Main constituents for S. oneidensis basal medium (SBM). Table S6. Main constituents for M9 buffer. [file 13068_2017_881_MOESM2_ESM.docx]

**Supporting tables**

**Table S1.** The reported electricity output by xylose-fed MFCs

| **Inoculum** | **Anode/**  **Cathode** | **MFC**  **volume** | **Power density**  **(mW/m^2^)** | **Carbon source** | **Reference** |
| --- | --- | --- | --- | --- | --- |
| a mixed bacterial culture that was originally enriched from domestic wastewater | Non-wet proofed carbon cloth / wet-proofed carbon cloths coated with platinum | 12ml | 2330 | 8mM  Xylose | [1] |
| a mixed bacterial culture | Graphite fiber brushes/ carbon cloth cathode coated with Pt and Plexiglas plate | 770ml | 673 | 20 mM Xylose | [2] |
| a mixed bacterial culture | Carbon  paper / Potassium ferricyanide with a continuous aeration | 50ml | 6.3 | 9.7 mM  Xylose | [3] |
| a synthetic microbial consortium composed of recombinant *E. coli* and *S. oneidensis* | Carbon  cloth/ Potassium ferricyanide | 140ml | 728.6 | 20 mM Xylose | [4] |

**Table S2.** The genes used in this study

| **Gene** | **Enzyme** | **E.C** | **Source** |
| --- | --- | --- | --- |
| *xylA* | xylose isomerase | 5.3.1.5 | *Escherichia coli* |
| *xylB* | xylulokinase | 2.7.1.17 | *Escherichia coli* |
| *XYL1* | xylose reductase | 1.1.1.307 | *Scheffersomyces stipitis* |
| *XYL2* | xylitol  dehydrogenase | 1.1.1.9 | *Scheffersomyces stipitis* |
| *XKS1* | xylulokinase | 2.7.1.17 | *Scheffersomyces stipitis* |
| *gxf1* | glucose/xylose facilitator1 |  | *Candida intermedia* |
| *xylT* | xylose-proton symporter |  | *Clostridium acetobutylicum* |

**Table S3.** Synthesized sequences of genes in this study

| *Gfx1* | ATGTCTCAAGATTCTCACTCTTCTGGTGCTGCTACTCCAGTTAACGGTTCTATCTTAGAAAAAGAAAAAGAAGATTCTCCAGTTTTACAAGTTGATGCTCCACAAAAAGGTTTCAAAGATTACATCGTTATCTCTATCTTCTGTTTCATGGTTGCTTTCGGTGGTTTCGTTTTCGGTTTCGATACTGGTACTATCTCTGGTTTCGTTAACATGTCTGATTTTAAGGATAGGTTCGGTCAACACCATGCTGATGGTACTCCATACTTATCTGATGTTCGTGTTGGTTTAATGATCTCTATCTTCAACGTTGGTTGTGCTGTTGGTGGTATCTTCTTATGTAAAGTTGCTGATGTTTGGGGTCGTCGTATCGGTTTAATGTTCTCTATGGCTGTTTACGTTGTTGGTATCATCATCCAAATCTCTTCTTCTACTAAATGGTACCAATTCTTCATCGGTCGTTTAATCGCTGGTTTAGCTGTTGGTACTGTTTCTGTTGTTTCTCCATTATTCATCTCTGAAGTTTCTCCAAAACAAATCCGTGGTACTTTAGTTTGTTGTTTCCAATTATGTATCACTTTAGGTATCTTCTTAGGTTACTGTACTACTTACGGTACTAAAACTTACACTGATTCTCGTCAATGGCGTATCCCATTAGGTTTATGTTTCGCTTGGGCTATCTTATTAGTTGTTGGTATGTTAAACATGCCAGAATCTCCACGTTACTTAGTTGAAAAACACCGTATCGATGAAGCTAAACGTTCTATCGCTCGTTCTAACAAAATCCCAGAAGAAGATCCATTCGTTTACACTGAAGTTCAATTAATCCAAGCTGGTATCGAACGTGAAGCTTTAGCTGGTCAAGCTTCTTGGAAAGAATTAATCACTGGTAAACCAAAAATCTTCCGTCGTGTTATCATGGGTATCATGTTACAATCTTTACAACAATTAACTGGTGATAACTACTTCTTCTACTACGGTACTACTATCTTCCAAGCGGTAGGTCTCAAAGATTCTTTTCAAACTTCTATCATCTTAGGTATCGTTAACTTCGCTTCTACTTTCGTTGGTATCTACGTTATAGAGCGTCTCGGTCGTCGTCTCTGTTTATTAACTGGTTCTGCTGCTATGTTCATCTGTTTCATCATCTACTCTTTAATCGGTACTCAACACTTATACAAACAAGGTTACTCTAACGAAACTTCTAACACTTACAAAGCTTCTGGTAACGCTATGATCTTCATCACTTGTTTATACATCTTCTTCTTCGCTTCTACTTGGGCTGGTGGTGTTTACTGTATCATCTCTGAATCTTACCCATTACGTATCCGTTCTAAAGCTATGTCTATCGCTACTGCTGCTAACTGGTTATGGGGTTTCTTAATCTCTTTCTTCACTCCATTCATCACTTCTGCTATCCACTTCTACTACGGTTTCGTTTTCACTGGTTGTTTAGCTTTCTCTTTCTTCTACGTTTACTTCTTCGTTTACGAAACTAAAGGTTTATCTTTAGAAGAAGTTGATGAAATGTACGCTTCTGGTGTTTTACCATTAAAATCTGCTTCTTGGGTTCCACCAAACTTAGAACACATGGCTCACTCTGCTGGTTACGCTGGTGCTGATAAAGCTACTGATGAACAAGTTTAA |
| --- | --- |
| *XYL1* | GCAGAATTCGCGGCCGCTTCTAGAGTACTAGAGAAAGAGGAGAAATACTAGATGGCTATCAAAATCGGTATCAACGGTTTCGGTCGTATGGGTCGTTTAGCTTTACGTGCTGCTTGGGATTGGGATGAAGTTGAATTTGTTCAAATCAACGATCCAGCTGGCGACGCTCACACTCTCGCTCACTTACTCGAATTTGATTCTGTTCACGGTCGTTGGCGTTACCCAGTTACTGCTAACGCTGATGCTATCCAAATCCAAGATAAAACTATCCGTACTACTCGTAACAAAGCTATCGCTGATACTGATTGGTCTGGTTGTGATGTTGTTATCGAAGCTTCTGGTGTTATGAAAACTAAAGCTTTATTACAAGCTTACTTAGATCAAGGTGTTAAACGTGTTGTTGTTACTGCTCCAGTTAAAGAAGATGGTGTTTTAAACGTTGTTATGGGTGTTAACCACCAATTATACGATCCAGCTATGCACCCAATCGTTACTGCTGCTTCTTGTACTACTAACTGTTTAGCTCCAGTTGTTAAAGTTATCCACGAACAAATCGGTATCAAACACGGTTCTATGACTACTATCCACGATATCACTAACACTCAAACTATCTTAGATGCTCCACACAAAGATTTACGTCGTGCTCGTGCTTGTGGTTTATCTTTAATCCCAACTACTACTGGTTCTGCTACTGCTATCACTCACATCTTCCCAGAATTAAAAGGTAAATTAAACGGTCACGCTGTTCGTGTTCCATTAGCTAACGCTTCTTTAACTGATTGTGTTTTCGAATTAGAACGTGCTGTTACTGAAGCTGAAGTTAACGCTTTATTAAAAACTGCTGCTGAAGGTGAATTAAAAGGTATCTTAGGTTACGAAGAACGTCCATTAGTTTCTGTTGATTACAAAACTGATCCACGTTCTTCTATCGTTGATGCTTTATCTACTATGGTTATCAACGGTACTCAATTAAAATTATACGTTTGGTACGATAACGAATGGGGTTACGCTAACCGTACTGCTGAACTCGCGAGGTTAGTTGGTCAACTCGATTTACCACGTTAATACTAGAGTACTAGTAGCGGCCGCCTGCAGG |
| *XYL2* | ATGACTGCTAACCCATCTTTAGTTTTAAACAAAATCGATGATATCTCTTTCGAAACTTACGATGCTCCAGAAATCTCTGAACCAACTGATGTTTTAGTTCAAGTTAAAAAAACTGGTATCTGTGGTTCTGATATCCACTTCTACGCTCACGGTCGTATCGGTAACTTCGTTTTAACTAAACCAATGGTTTTAGGTCACGAATCTGCTGGTACTGTTGTTCAAGTTGGTAAAGGTGTTACTTCTTTAAAAGTTGGTGATAACGTTGCTATCGAACCAGGTATCCCATCTCGTTTCTCTGATGAATACAAATCTGGTCACTACAACTTATGTCCACACATGGCTTTCGCTGCTACTCCAAACTCTAAAGAAGGTGAACCAAACCCACCAGGTACTTTATGTAAATACTTCAAATCTCCAGAAGATTTCTTAGTTAAATTACCAGATCACGTTTCTTTAGAATTAGGTGCTTTAGTTGAACCATTATCTGTTGGTGTTCACGCTTCTAAATTAGGTTCTGTTGCTTTCGGTGATTACGTTGCTGTTTTCGGTGCTGGTCCAGTTGGTTTATTAGCTGCTGCTGTTGCTAAAACTTTCGGTGCTAAAGGTGTTATCGTTGTTGATATCTTCGATAACAAATTAAAAATGGCTAAAGATATCGGTGCTGCTACTCACACTTTCAACTCTAAAACTGGTGGTTCTGAAGAATTAATCAAAGCTTTCGGTGGTAACGTTCCAAATGTTGTACTGGAATGTACTGGTGCTGAACCATGTATCAAATTAGGTGTTGATGCTATCGCTCCAGGTGGTCGTTTCGTTCAAGTTGGTAACGCTGCTGGTCCAGTTTCTTTCCCAATCACTGTTTTCGCTATGAAAGAATTAACTTTATTCGGTTCTTTCCGTTACGGTTTCAACGATTACAAAACTGCTGTTGGTATCTTCGATACTAACTACCAAAACGGTCGTGAAAACGCTCCAATCGATTTCGAACAATTAATCACTCACCGTTACAAATTCAAAGATGCTATCGAAGCTTACGATTTAGTTCGTGCTGGTAAAGGTGCTGTTAAATGTTTAATCGATGGTCCAGAATAA |
| *XKS1* | ATGACTACTACTCCATTCGATGCTCCAGATAAATTATTCTTAGGTTTCGATTTATCTACTCAACAATTAAAAATCATCGTTACTGATGAAAACTTAGCTGCTTTAAAAACTTACAACGTTGAATTTGATTCTATCAACTCTTCTGTTCAAAAAGGTGTTATCGCTATCAACGATGAAATCTCTAAAGGTGCTATCATCTCTCCAGTTTACATGTGGTTAGATGCTTTAGATCACGTTTTCGAAGATATGAAAAAAGATGGTTTCCCATTCAACAAAGTTGTTGGTATCTCTGGTTCTTGTCAACAACACGGTTCTGTTTACTGGTCTCGTACTGCTGAAAAAGTTTTATCTGAATTAGATGCTGAATCTTCTTTATCTTCTCAAATGCGTTCTGCTTTCACTTTCAAACACGCTCCAAACTGGCAAGATCACTCTACTGGTAAAGAATTAGAAGAATTTGAACGTGTTATCGGTGCTGATGCTTTAGCTGATATCTCTGGTTCTCGTGCTCACTACCGTTTCACTGGTTTACAAATCCGTAAATTATCTACTCGTTTCAAACCAGAAAAATACAACCGTACTGCTCGTATCTCTTTAGTTTCTTCTTTCGTTGCTTCTGTTTTATTAGGTCGTATCACTTCTATCGAAGAAGCTGATGCTTGTGGTATGAACTTATACGATATCGAAAAACGTGAATTTAACGAAGAATTATTAGCTATCGCTGCTGGTGTTCACCCAGAATTAGATGGTGTTGAACAAGATGGTGAAATCTACCGTGCTGGTATCAACGAATTAAAACGTAAATTAGGTCCAGTTAAACCAATCACTTACGAATCTGAAGGTGATATCGCTTCTTACTTCGTTACTCGTTACGGTTTCAACCCAGATTGTAAAATCTACTCTTTCACTGGTGATAACTTAGCTACTATCATCTCTTTACCATTAGCTCCAAACGATGCTTTAATCTCTTTAGGTACTTCTACTACTGTTTTAATCATCACTAAAAACTACGCTCCATCTTCTCAATACCACTTATTCAAACACCCAACTATGCCAGATCACTACATGGGTATGATCTGTTACTGTAATGGCAGCTTAGCTCGTGAAAAGGTTCGTGATGAAGTTAACGAAAAATTCAACGTTGAAGATAAAAAATCTTGGGATAAATTCAACGAAATCTTAGATAAATCTACTGATTTCAACAACAAATTAGGTATCTACTTCCCATTAGGTGAAATCGTTCCAAACGCTGCTGCTCAAATCAAACGTTCTGTTTTAAACTCTAAAAACGAAATCGTTGATGTTGAATTAGGTGATAAAAACTGGCAACCAGAAGATGATGTTTCTTCTATCGTTGAATCTCAAACTTTATCTTGTCGTTTACGTACTGGTCCAATGTTATCTAAATCTGGTGATTCTTCTGCTTCTTCTTCTGCTTCTCCACAACCAGAAGGTGATGGTACTGATTTACACAAAGTTTACCAAGATTTAGTTAAAAAATTCGGTGATTTATACACTGATGGTAAAAAACAAACTTTCGAATCTTTAACTGCTCGTCCAAACCGTTGTTACTACGTTGGTGGTGCTTCTAACAACGGTTCTATCATCCGTAAAATGGGTTCTATCTTAGCTCCAGTTAACGGTAACTACAAAGTTGATATCCCAAACGCTTGTGCTTTAGGTGGTGCTTACAAAGCTTCTTGGTCTTACGAATGTGAAGCTAAAAAAGAATGGATCGGTTACGATCAATACATCAACCGTTTATTCGAAGTTTCTGATGAAATGAACTTATTCGAAGTTAAAGATAAATGGTTAGAATACGCTAACGGTGTTGGTATGTTAGCTAAAATGGAATCTGAATTAAAACACTAA |
| *xylT* | ATGAACAAAAAAATCTCTCCAGCTTTAATCTACTTCTTCGGTGCTTTCGGTGGTTTCATGTTCGGTTACGATATCGGTATCATAAATGGTGCTCTCCCAGGTATCAATGCGACTTGGCACGTGAGCTCTTGGTTAGAAGGTTTCATCACTTCTGGTTTATTCGTTGGTGCTATGATCGGTGCTTCTTTAATGGCTTCTTTAGCTGATCGTTTCGGTCGTCGTCGTATGATCATGTGGTCTGCTATCGTTTTCGCTTTAGGTGCTTTAGGTTCTGCTGTTTCTACTTCTACTAACTTATTAATCGGTGCTCGTGTTATCTTAGGTGTTGCTGTTGGTGGTGCTTCTGCTTTAGTTCCAATGTACATGGGTGAAATCTCTCCAGCTGAAACTCGTGGTAAATTATCTGGTTTAAACCAATTAATGATCACTGTTGGTATGTTATTCTCTTACGGTGTTAACTTCGCTTTCGCTGGTGCTTTCGAAGGTTGGCGTTGGATGTTAGGTGGTGCTATGGTTCCAGCTATGGTTTTATTAATCGGTACTTTCATCTTACCAGAATCTCCACGTTTCTTAGCTCGTATCGGTAAAACTGAATTAGCTAAACAAGTTCTCCAGACTCTCCGTTCTAAAGAGGAAGCTGAAACTGAATACCAAGAAATCATCAACTCTAAACACACTGAAACTGGTTCGTTTGGTGACTTATTCGCGAAGCAGGCTCTCCCAGCTGTTATAGCGGGCTGTGGCTTAACTTTATTACAGCAAATCCAAGGTGCTAACACTATCTTCTACTACTCTTCTCAAATCTTATCTAACGTTTTCGGTTCTGCTAACGGTGGTACTATCTCTACTGTTGGTATCGGTGTTGTTTTAGTTTTAGCTACTATCGTTACTTTATTAGTTGTTGATAAATTCAAACGTCGTACTTTATTCATGACTGGTTCTATCGGTATGGGTGCTTCTTTATTATTAGTTGGTTTAATCTACCCATACTCTGAAGCTAAACACGCTTGGGCTACTTGGTTAGTTTTCTTCTTCATCTGTTTATACGTTGTTTTCTACGCTTACTCTTGGGCTGCTACTACTTGGATCGTTGTTGGTGAATTATTCCCATCTAACGTTCGTGGTTTAGCTACTGGTATCGCTTCTGCTGTTAACTGGTTCGGTAACATCTTAGTTGCTTTATTCTTCCCAGTTCTCTTAGAAACTGTTGGCTTATCTGTTATCTTTTTCGGTTTCGCTGCTATCTGTATCATCGGTTTCTTATTCGCTAAATACGTTTTATACGAAACTAAAGGTAAATCTTTAGAAGAAATCGAAACTTACTTATACAACCGTTCTATCGGTAAAGTTCGTGGTTTAAACGAATAA |
| *xylA* | ATGCAAGCTTACTTCGATCAATTAGATCGTGTTCGTTACGAAGGTTCTAAATCTTCGAACCCATTAGCGTTCCGTCACTACAATCCAGATGAATTAGTTTTAGGTAAACGTATGGAAGAACACTTACGTTTCGCTGCTTGTTACTGGCACACTTTCTGTTGGAACGGTGCTGATATGTTCGGTGTTGGTGCTTTCAACCGTCCATGGCAACAACCAGGTGAAGCTTTAGCTTTAGCTAAACGTAAAGCTGATGTTGCTTTCGAATTTTTCCACAAATTACACGTTCCATTCTACTGTTTCCACGATGTTGATGTTTCTCCAGAAGGCGCGAGCTTAAAAGAATACATAAACAACTTCGCTCAAATGGTTGATGTTTTAGCTGGTAAACAAGAAGAATCTGGTGTTAAATTATTATGGGGTACTGCTAACTGTTTCACTAACCCACGTTACGGTGCTGGTGCTGCTACTAACCCAGATCCAGAAGTTTTCTCTTGGGCTGCTACTCAAGTTGTTACTGCTATGGAAGCTACTCACAAATTAGGTGGCGAGAACTACGTACTGTGGGGTGGTCGTGAAGGCTACGAAACATTATTAAACACTGACTTACGTCAAGAACGTGAACAATTAGGTCGTTTCATGCAAATGGTTGTTGAACACAAACACAAAATCGGTTTCCAAGGTACTTTATTAATCGAACCAAAACCACAAGAACCAACTAAACACCAATACGATTACGATGCTGCTACTGTTTACGGTTTCTTAAAACAATTCGGTTTAGAAAAAGAAATCAAATTAAACATCGAAGCTAACCACGCTACTTTAGCTGGTCACTCTTTCCACCACGAAATCGCTACTGCTATCGCTTTAGGTTTATTCGGTTCTGTTGATGCTAACCGTGGTGATGCTCAATTAGGTTGGGATACTGATCAATTCCCAAACTCTGTTGAAGAAAACGCTTTAGTTATGTACGAAATCTTAAAAGCTGGTGGTTTCACAACTGGTGGTCTCAACTTCGATGCGAAAGTTCGTCGTCAATCTACTGATAAATACGATTTATTCTACGGTCACATCGGTGCGATGGATACAATGGCTCTCGCTTTAAAAATAGCTGCTCGTATGATCGAAGATGGTGAATTAGATAAACGTATCGCTCAACGTTACTCTGGTTGGAACTCTGAATTAGGTCAACAAATCTTAAAAGGTCAAATGTCTTTAGCTGATTTAGCTAAATACGCTCAAGAACACCACTTATCTCCAGTTCACCAATCTGGTCGTCAAGAACAATTAGAAAACTTAGTTAACCACTACTTATTCGATAAATAA |
| *xylB* | ATGTACATCGGTATCGATTTAGGTACTTCTGGTGTTAAAGTTATCTTATTAAACGAACAAGGTGAAGTTGTTGCTGCTCAAACTGAAAAATTAACTGTTTCTCGTCCACACCCATTATGGTCTGAACAAGATCCAGAACAATGGTGGCAAGCTACTGATCGTGCTATGAAAGCTTTAGGTGATCAACACTCTTTACAAGATGTTAAAGCTTTAGGTATCGCTGGTCAAATGCACGGTGCTACTTTATTAGATGCTCAACAACGTGTTTTACGTCCAGCTATCTTATGGAACGATGGTCGTTGTGCTCAAGAATGTACTTTATTAGAAGCTCGTGTTCCACAATCTCGTGTTATCACTGGTAATTTAATGATGCCAGGCTTCACTGCTCCAAAGTTATTATGGGTTCAACGTCACGAACCAGAAATCTTCCGTCAAATCGATAAAGTTTTATTACCAAAAGATTACTTACGTTTACGTATGACTGGTGAATTTGCTTCTGATATGTCTGATGCTGCTGGTACTATGTGGTTAGATGTTGCTAAACGTGATTGGTCTGATGTTATGTTACAAGCTTGTGATTTATCTCGTGATCAAATGCCAGCTTTATACGAAGGTTCTGAAATCACTGGTGCTTTATTACCAGAAGTTGCTAAAGCTTGGGGTATGGCTACTGTTCCAGTTGTTGCTGGTGGTGGTGATAACGCTGCTGGTGCTGTTGGTGTTGGTATGGTTGATGCTAACCAAGCTATGTTATCTTTAGGTACTTCTGGTGTTTACTTCGCTGTTTCTGAAGGTTTCTTATCTAAACCAGAATCTGCTGTTCACTCTTTCTGTCACGCTTTACCACAACGTTGGCACTTAATGTCTGTTATGTTATCTGCTGCTTCTTGTTTAGATTGGGCTGCTAAATTAACTGGTTTATCTAACGTTCCAGCTTTAATCGCTGCTGCTCAACAAGCTGATGAATCTGCTGAACCAGTTTGGTTCTTACCATACTTATCTGGTGAACGTACTCCACACAACAACCCACAAGCTAAAGGTGTTTTCTTCGGTTTAACTCACCAACACGGTCCAAACGAATTAGCTCGTGCTGTTTTAGAAGGTGTTGGTTACGCTTTAGCTGATGGTATGGATGTTGTTCACGCGTGTGGTATCAAACCACAATCGGTTACTCTCATCGGTGGTGGCGCTCGTTCTGAATACTGGCGTCAAATGTTAGCTGATATCTCTGGTCAACAATTAGATTACCGTACTGGTGGTGATGTTGGCCCCGCTCTCGGTGCTGCTCGTTTAGCTCAAATCGCTGCTAACCCAGAAAAATCTTTAATCGAATTATTACCACAATTACCATTAGAACAATCTCACTTACCAGATGCTCAACGTTACGCTGCTTACCAACCACGTCGTGAAACTTTCCGTCGTTTATACCAACAATTATTACCATTAATGGCTTAA |

**Table S4.** The **s**trains and plasmids used in this study

| **Strain or plasmid** | **Feature(s)** | **Source or reference** |
| --- | --- | --- |
| *S. oneidensis* |  |  |
| MR-1 | Wild-type strain | Our Lab[[5](#_ENREF_1)] |
| WT | carrying pYYDT | Our Lab[[5](#_ENREF_1)] |
| XE | carrying pYYDT-XE | This study |
| GE | carrying pYYDT-GE | This study |
| XS | carrying pYYDT-XS | This study |
| GS | carrying pYYDT-GS | This study |
| *E. coli* |  |  |
| Trans T1 | F-80(lacZ)ΔM15ΔlacX74hsdR(rk -, mk +)ΔrecA1398endA1tonA | Transgen Biotech |
| Plasmids |  |  |
| pYYDT | 5.9kb; Km^r^; *lacZ* | Our Lab[[5](#_ENREF_1)] |
| pYYDT-XE | Plasmid with the *xylT*, *xylA* and *XylB* gene inserted | This study |
| pYYDT-GE | Plasmid with the *Gxf1*, *xylA* and *XylB* gene inserted | This study |
| pYYDT-XS | Plasmid with the *xylT*, *XYL1*, *XYL2 and XKS1* gene inserted | This study |
| pYYDT-GS | Plasmid with the *Gxf1*, *XYL1*, *XYL2 and XKS1* gene inserted | This study |

**Table S5. The m**ain constituents of the *S. oneidensis* basal medium (SBM) [[6](#_ENREF_2), [7](#_ENREF_3)]

| Main constituent | Concentration |
| --- | --- |
| K_2_HPO_4_ | 0.225 g/l |
| KH_2_PO_4_ | 0.225 g/l |
| NaCl | 0.46 g/l |
| (NH_4_)_2_SO_4_ | 0.225 g/l |
| MgSO_4_ | 0.117 g/l |
| Casamino acids | 0.2 g/l |
| Amino acid mix | 10 ml/l |
| Trace mineral solution | 10 ml/l |

The *Shewanella* basal medium (SMB) supplemented xylose formed a synthetic medium, which had well-defined ingredients and was mainly used to study the growth and xylose consumption of *Shewanella* strains [[6](#_ENREF_2), [7](#_ENREF_3)]. We thus used the SMB+xylose to examine the cell growth and xylose utilization.

**Table S6. The** main constituents of the M9 buffer [[8](#_ENREF_4)]

| Main constituent | Concentration |
| --- | --- |
| Na_2_HPO_4_ | 6 g/l |
| KH_2_PO_4_ | 3 g/l |
| NaCl, | 0.5 g/l |
| NH_4_Cl | 1 g/l |
| MgSO_4_ | 1 mM |
| CaCl_2_ | 0.1 mM |

The M9 buffer supplemented with xylose and 5% LB constituted the M9 medium, which included abundant nutrients and was generally used to study the MFC performance of prolonged power generation by *S. oneidensis*. 5% LB included many amino acids and vitamins to maintain the viability of *Shewanella* to enable a stable power generation. It was a generally used medium to study electricity generation in the *Shewanella*-based MFCs [8].

**References**

[1]. Huang L, Logan BE. Electricity production from xylose in fed-batch and continuous-flow microbial fuel cells. Appl Microbiol Biotechnol. 2008; 80(4):655-64.

[2]. Huang L, Zeng RJ, Angelidaki I. Electricity production from xylose using a mediator-less microbial fuel cell. Bioresour Technol. 2008; 99(10):4178-84.

[3]. Catal T, Li K, Bermek H, Liu H. Electricity production from twelve monosaccharides using microbial fuel cells. J Power Sources. 2008; 175(1):196-200.

[4]. Yang Y, Wu Y, Hu Y, Cao Y, Poh CL, Cao B, Song H. Engineering electrode-attached microbial consortia for high-performance xylose-fed microbial fuel cell. ACS Catal. 2015; 5(11):6937-45.

[5]. Yang Y, Ding Y, Hu Y, Cao B, Rice SA, Kjelleberg S, Song H. Enhancing Bidirectional Electron Transfer of *Shewanella oneidensis* by a Synthetic Flavin Pathway. ACS Synth Biol. 2015; 4(7):815-23.

[6]. Flynn CM, Hunt KA, Gralnick JA, Srienc F. Construction and elementary mode analysis of a metabolic model for *Shewanella oneidensis* MR-1. Biosystems. 2012; 107(2):120-28.

[7]. Kouzuma A, Meng XY, Kimura N, Hashimoto K, Watanabe K. Disruption of the putative cell surface polysaccharide biosynthesis gene SO3177 in *Shewanella oneidensis* MR-1 enhances adhesion to electrodes and current generation in microbial fuel cells. Appl Environ Microbiol. 2010; 76(13):4151-7.

[8]. Yong YC, Yu YY, Zhang X, Song H. Highly active bidirectional electron transfer by a self-assembled electroactive reduced-graphene-oxide-hybridized biofilm. Angew Chem Int Ed. 2014; 53(17):4480-83.
